# The Experience and Enlightenment of the Community-Based Long-Term Care in Japan

**DOI:** 10.3390/healthcare10091599

**Published:** 2022-08-23

**Authors:** Yun-Ru Zhou, Xiao Zhang

**Affiliations:** School of Public Health, Southeast University, Nanjing 210009, China

**Keywords:** long-term care, community care, preventive service, development experience

## Abstract

(1) Background: China’s population aging situation is severe, but the construction of the long-term care insurance system is still in its infancy. Through summarizing the long-term care experience in Japan, this paper explores the suggestions for the development of long-term care in China. (2) Methods: Based on literature research and policy review, we sorted out the relevant practices and safeguard measures of the long-term care insurance system in Japan, and summarized the characteristics of Japanese community care. (3) Results: In the development of long-term care services, Japan has gradually established a multi-level, systematic, and precise elderly care service model. Its community care has the characteristics of policy support, intensive intervention, complete elements, and strict evaluation. China’s long-term care services should learn from Japan’s experience, strengthen institutional guarantees, improve relevant supporting policies, encourage multiple subjects to participate in community care based on integrating community resources, and establish community care evaluation mechanism.

## 1. Introduction

In recent years, China’s population aging situation is severe. According to the “China Statistical Yearbook 2021”, as of 2020, the number of people aged 65 and over in China has reached 190.64 million [1]. A prediction study by Xiamen University on the scale of disability among the elderly in urban and rural areas in China shows that the total number of disabled elderly in China will rapidly increase from 43.75 million in 2020 to 91.4 million in 2050 [2]. The process of disability and dementia among the elderly in China is accelerating. With the progress of the economy, people have also put forward higher requirements for social security welfare. The demand for care services for disabled and dementia people has also given birth to China’s long-term care insurance (LTCI). In 2016, China officially started the pilot work of LTCI and set up 15 pilot cities. In 2020, the number of pilot cities increased to 49. The National Medical Insurance Administration has also issued a series of guidance documents, policies, and regulations. However, China is still in the initial stage of promoting the construction and development of LTCI at the national level, and there are still many problems that need to be further solved and clarified.

“The 14th Five-Year Plan for National Economic and Social Development of the People’s Republic of China and the Outline of the Vision for 2035” (2022) put forward the development goal of “building an elderly care service system coordinated by home and community institutions and combining medical care and health care” [3]. Before this, Sun Juanjuan (2021) had already proposed that it is necessary to build a multi-level care service system with families, communities, and institutions as the main body, expand the coverage of services, and make services penetrate into families and communities [4]. However, from the practices of the pilot cities, it can be seen that the long-term care services currently provided are home care and institutional care, and few cities independently carry out community care [5]. Xing Yuzhou (2021) believes that the current community elderly care service system in China not only needs to be improved in terms of the management system, but also faces problems such as a shortage of service personnel and insufficient specialization [6]. Therefore, China’s current main task is to explore how to develop community care.

Long-term care (LTC) was derived from the report entitled “Establishing an International Consensus on Long-Term Care Policies for the Elderly” which was reported by the World Health Organization in 2000. It refers to a system of activities carried out by informal providers of caregivers (family, friends, and/or neighbors) and professionals (health, social, and others), which can ensure that people with limited self-care skills can maintain the highest possible quality of life based on individual preferences choice and enjoy the maximum possible independence, autonomy, participation, personal fulfillment, and human dignity [7]. Countries around the world usually provide basic living care or funds/service guarantees for medical care closely related to basic life for such people in the form of establishing LTCI [8].

LTCI was first developed in the United States around the 1980s and was introduced to Asia around 2000. Germany was the first country in the world to formally legislate public LTCI (1995) [9], while Japan was the first Asian country to establish public LTCI. LTCI in the United States is mainly composed of public security plans (Medicare, Medicaid) and commercial LTCI [10]. According to the research of Jing Tao and Yang Shu (2018), Medicare cannot provide real long-term care services or meet the long-term care needs of the disabled elderly. In addition, Medicaid plays a pivotal role in the long-term care system for the elderly in the United States, but it has strict restrictions, mainly for low-income individuals or families [11]. Germany decides whether to participate in public LTCI or commercial LTCI according to the income status of its citizens. Its LTC services include home care, partial institutional care, and full institutional care [10]. LTCI in Japan is compulsory for all citizens over the age of 40, and the insurance funds are jointly raised by social insurance and taxation [12]. It provides home care, institutional care, and community care.

Many Chinese scholars believe that China can learn from Japan’s experience. Liu Xiaomei and other scholars (2019) believe that China has a rapidly increased demand for long-term care in an aging society which is similar to Japan, so we can draw inspiration from Japan’s experience [13]. Zhao Jianguo and Shao Siqi (2019) both believe that Japan entered an aging society 30 years earlier than China, and Japan’s experience in the construction of the elderly care system is very important for China to “completely build a home-based, community-based, institution-supported elderly care service system with complete functions, moderate scale, and coverage of urban and rural areas” [14]. Japan adopted a complete public LTCI model, the beneficiaries are only limited by age, not income level, and the development of community care is particularly prominent. The LTCI system currently being piloted in China is similar to that in Japan. In addition, because Japan and China both belong to East Asian countries, they are relatively similar in terms of physiological characteristics, living habits, cultural heritage, and social welfare concepts. Therefore, this paper takes Japan’s LTCI system as the research object and mainly summarizes the development process of long-term care in Japan, especially community care, summarizes its characteristics, and puts forward suggestions for the development of community care in China.

## 2. Materials and Methods

The English literature was searched in PubMed with the phrases “long-term care” or “long-term care insurance” or “Community care” and “Japan” in the title or abstract. The articles that met the criteria were screened out by reading the title and abstract one by one, and the full text was obtained from literature databases and platforms such as PubMed, Medline, and Google Scholar. Chinese literature searched in literature databases such as China Journal Full-text Database (CNKI), Wanfang Data Knowledge Service Platform, etc. The abstract had to contain “long-term care insurance” or “community care” and “Japan”. We selected articles that met the criteria by reading the title and abstract one by one, and read the full text selectively. The literature search period was from 2000 to 2021. At the same time, we browsed Japanese and Chinese government and agency websites related to LTCI to collect the required information. The data retrieval period ended in 2021. The data retrieval period ended at the end of 2021.

Inclusion criteria: We included relevant literature on LTCI policies or relevant laws and regulations.

Exclusion criteria: Literature without clear source; literature that mentions the name of the national (or regional) long-term care insurance policy, but lacks a description of the specific measures; literature that is published repeatedly.

The number of articles that were found in the initial search was 1378 (including 1258 English literature and 120 Chinese literature). The following types of documents were not included: Clinical trial, meta-analysis, randomized controlled trial, meeting abstract, editorial, proceedings paper, and other types such as opinions or comments (189 articles in total). According to the inclusion criteria and exclusion criteria, the final number of articles that were included in this paper was 28 (3 books not included).

Based on the literature research and policy review, this paper sorted out the safeguards of Japan’s LTCI system, focused on the development of Japanese community care, and summarized the characteristics of Japanese community care. At the same time, based on the actual situation in China, it put forward relevant suggestions for the further development of community care in China.

## 3. Results

### 3.1. Long-Term Care Insurance in Japan

As one of the earliest Asian countries to establish the LTCI system, Japan has established a relatively complete care service system [15]. It is backed by law, has defined management departments, beneficiaries, finance sources, and care levels, and has rigorous care needs assessments, care market access, and supervision. The relevant information on its long-term care insurance system is shown in Table 1.

With the rapid change in the population structure and the disintegration of traditional family structures, in response to the expected shift from traditional home care to social care, the Japanese government took steps in the mid-1990s to promote the “socialization of care” for the elderly. In 2000, based on the “Long-Term Care Insurance Act”, Japan launched the LTCI system to reduce the burden on family caregivers [16]. The purpose was to determine whether the elderly need to be cared for according to their physical conditions, and to provide corresponding care services according to the assessed care level.

The beneficiaries are divided into two categories: Category I beneficiaries are the elderly aged 65 years and above, and category II beneficiaries are people aged 40–64 years with disabilities. There are seven care levels, including two requiring support (levels 1 and 2) and five requiring long-term care (levels 1–5).

Municipalities, as insurance providers to the LTCI system, are responsible for implementing the LTC program and determining insurance premiums by measuring the balance between the needs of the population and the number of services available in the area. Half of the LTC service fees come from taxes (25% from the central government, 12.5% from the prefectures, and 12.5% from the municipalities) and half comes from premium contributions.

The Long-Term Care Insurance Act has been periodically revised every three years. The reforms in Japan’s long-term care insurance are shown in Table 2. Due to an aging society, the LTCI has been facing escalating costs and recent reforms focus on cost containment, while keeping the quality and quantity of long-term care services.

Since the implementation of this system, the effect of LTC services has been obvious, and the system has been continuously improved. The mature LTCI system has laid the foundation for the formation of a community-based, small-scale, multi-functional elderly care service model in Japan [17].

### 3.2. Community Care in Japan

#### 3.2.1. Development History

With the increase in the unmarried population, the acceleration of urbanization, and the increase in single-parent families or families with separated parents and children, the number of elderly people living alone in Japan is increasing [18]. In order to cope with the situation of rapid aging, after the implementation of LTCI, the construction of regional comprehensive services was strengthened, and it was proposed that community medical services should be effectively connected with LTC services, and the integrated community care system (ICCS) has been implemented since 2006. This system was designed to provide (1) medical care, (2) long-term care, (3) long-term preventive care, (4) living support, and (5) housing services collaboratively within a 30-min daily walk life circle (the ideal range for each community) [19]. This system is managed by municipal governments, using a fund from the LTCI system.

In 2012, the Japanese government launched the “Amendment to the Long-term Care Insurance Act to Strengthen Long-term Care Insurance Services”, which clearly proposed to support the insured’s sustainable life in the community environment where they are used to, and to integrate the originally independent living support and medical services. It is committed to building a community-based integrated care system that integrates medical care, long-term care, long-term prevention care, and living support and housing service [15], emphasizing the care concepts of self-help, mutual assistance, and public assistance.

In 2015, the “Long-term Care Insurance Act” was revised again, and some supplements were made: All care expenses (including home care) were handed over to the municipal community assistance centers for management; the community assistance services were enriched, home medical care was promoted, and a nursing prevention cooperation mechanism was implemented; community comprehensive care service seminars were to be regularly held; and it was stipulated that the occupancy level of special nursing care institutions should be above level 3, and further play the role of community elderly care functions and other related content [20].

In 2017, Japan incorporated disability services into community comprehensive care services to provide integrated, continuous/integrated services for the elderly and the disabled [21]. At present, the community has become the main carrier of social welfare such as pensions in Japan [22].

#### 3.2.2. Characteristic

##### Comprehensive and Systematic Policy Support

In addition to the above-mentioned laws and regulations, Japan has also successively released the “Research Report on Nursing for the Elderly” (Ministry of Health, Labour and Welfare Elderly Nursing Research Association, 2003), and the “Report on the Community Comprehensive Care Service System Research Association” (Community Comprehensive Care Service System Seminar, 2013). “Law for Comprehensive Protection of Community Medical and Care Services” (2014) and other policy documents and research reports, to escort the implementation of community care.

Regarding the qualifications and incentives of caregivers, the Law on Social Welfare and Nursing Welfare Workers was promulgated in 1987, and a national qualification certificate was issued. When the Long-term Care Insurance Act was implemented in 2000, a care broker (nursing support specialist) was established. In 2018, the “Law on Improving the Treatment of Nursing Practitioners to Ensure the Reserve of Nursing Practitioners and Others” was promulgated to increase remuneration and activate the nursing talent market.

Regarding the construction of elderly care facilities and the environment, policy documents such as “Guidelines for Designing Housing for a Longevity Society” (1995), “Law on Promoting the Mobility of the Elderly and Disabled” (2000), and other policy documents have been promulgated successively. Relevant standards and requirements have been formulated for residential construction and community planning to provide a more comfortable and convenient retirement environment for the elderly [15].

In addition, Japan has also made policy regulations on the content of care services such as volunteer management, and has built a systematic and comprehensive policy system with the “Long-term Care Insurance Act” as the core.

##### Intensive Intervention and Early Prevention

After 2006, elderly with mild disabilities were included in the coverage of LTCI, and delayed the onset or worsening of disability further deterioration of this population by providing early intervention services to this population. Care prevention services (Services that prevent or reduce disability and improve life skills [23]) are provided in ICCS, focusing on care prevention management, developing care plans primarily for “requiring support” level 1 and 2 persons, and carrying out preventive work for those who can take care of themselves, etc. [24].

From the current point of view, the development of early prevention services can improve the health of the elderly, increase the healthy life expectancy of the elderly, and improve the quality of life of the elderly. In the long run, early intervention and prevention can help reduce the financial pressure on LTCI.

##### Integrate Resources and Complete Elements

Since 2006, Japan has positioned its long-term care policy as community-based integrated care, and is committed to building ICCS. Each community-integrated care service center is “small-scale and multi-functional”. It provides services within a community, occupies a small area, and provides a variety of services. In addition to government-authorized care institutions and medical institutions, the subjects participating in the care service also include non-profit organizations, social workers, volunteers, and so on.

The ICCS includes five elements: “residence”, “medical care”, “long-term care”, “care prevention”, and “living support” [25]. “Residence” is the foundation; In addition to self-owned and rented houses, there are fully-equipped apartments for the elderly. “Medical care” refers to medical institutions, community hospitals, home medical care, visiting medical care, visiting rehabilitation, and other medical services. “Long-term care” includes institutional care and home care. “Care prevention” has been explained above. “Living support” includes the services provided by social workers and the support provided by volunteers from family and neighbors. The five elements are indispensable. They cooperate with each other to develop fragmented services into an integrated type and jointly maintain the normal operation of the ICCS [26].

##### Rigorous Evaluation and Continuous Improvement

The care work plan of ICCS in Japan is revised every three years and a strict evaluation will be carried out before revision. The PDCA (plan, do, check, action) cycle assessment is performed by using the “5W2H” (what, why, when, where, who, how, how much) assay.

The first step is to communicate with the client through the care meeting in the community to achieve information transparency and investigate the actual life and care needs of the elderly in the community, and then through quantitative analysis to find out the key points that the community needs to pay attention to and the society resource that needs to be explored. The second step is to share the situation, plans, and implementation results of each community through inter-community care conferences, which will help communities learn from each other to formulate more appropriate care plans. The third step is to discuss specific policies and formulate a final care plan and implement it strictly by the plan. Finally, the evaluation is carried out from multiple aspects during the implementation process, such as the service quality satisfaction evaluation of the service object, and the analysis and evaluation of the support status through the nursing support evaluation system.

At the same time, in order to ensure the quality of the care services, the government will also set up departments and agencies especially responsible for supervising care services, and promptly ban care service agencies with low service levels. Once the improper profit-making behavior of the care service institution is discovered, its qualification for care service will be canceled immediately.

Through the PDCA cycle evaluation mechanism, each community continuously improves service content and service quality, and then develops a community comprehensive care service center that conforms to the actual situation of each community.

### 3.3. The Enlightenment for China

#### 3.3.1. Strengthen Institutional Guarantees and Improve Supporting Policies

At present, China has proposed to strengthen the functional connection between the construction of community elderly care service facilities and other community services in policy documents [27], but there is a lack of specific legal provisions and implementation rules. With the continuous exploration and development of China’s LTCI system, we should seize the opportunity of the start-up of the LTCI system, combined with China’s national conditions and the experience of pilot cities, further improve the relevant system guarantees through legislation. At the same time, according to the actual situation, stipulate the scope of each functional organization to realize the breadth and continuity of services [3], and to form a scientific and sound LTCI system.

In addition, it is also necessary to accelerate the introduction of supporting policies related to LTCI, and improve and standardize the behavior of the care industry constantly, including the qualification and incentives for caregivers, the standardization of elderly care facilities and environments, supervision, and demand assessment systems, volunteers management and other aspects of management and constraints to ensure that care services have laws to follow and violations must be investigated.

#### 3.3.2. Optimize Community Care Service Resource Allocation

“Small-scale and multi-functional” community comprehensive care service centers can improve the utilization rate of care resources, provide convenient and fast services for care recipients, and effectively improve the satisfaction of care recipients. Therefore, various types of care service subjects should be encouraged and attracted to enter the community. We should integrate the elderly care service resources, and provide various types of services such as bed care, medical care, preventive education, and living assistance for care recipients in the community, to ensure that the care recipients can enjoy “residence”, “medical care”, “long-term care”, “care prevention”, and “living support” in the community.

At the same time, a community platform can be used to establish a coordination mechanism between care service agencies and care recipients, and help them formulate personalized care service plans according to their personal and family wishes. In addition, it is necessary to pay more attention to early mild symptoms. Through preventive education, regular screening, and early intervention, the development of severe diseases can be effectively avoided and the pressure on care funds can be reduced.

#### 3.3.3. Encourage Diverse Subjects to Participate in Community Care Services

In Japan’s community comprehensive care service centers, the main bodies involved in care services include government-authorized care institutions, medical institutions, and non-profit organizations, social workers, and volunteers. China should also form a pattern in which various subjects participate in community care services.

The most basic are professional care institutions and medical institutions. Professional care practitioners, doctors, and nurses provide corresponding care and medical services to care objects in the community. It is also possible to tap the social resources in the community and surrounding areas to provide assistance for the care services in the community, such as inviting social workers, non-governmental organizations, and non-profit organizations to regularly enter the community or even the homes of care recipients to provide health consultation, care needs surveys, life support, enrich entertainment life, and other services to alleviate the shortage of community care manpower. In addition, housewives, students, and elderly people in good health in the community can also be encouraged to participate, undertake part of the work of life support and psychological comfort, and formulate corresponding reward mechanisms, such as young people being included in individual volunteer hours, the elderly rely on payment in exchange for care services, etc.

#### 3.3.4. Establish an Appropriate Community Care Assessment Mechanism

Drawing on the evaluation mechanism of Japan’s ICCS, in addition to accepting supervision from the government, the community itself should also establish a service feedback and evaluation mechanism to ensure that the care recipients in the community can get better services. For example, regular assessments on the entities providing care services should be conducted, and institutions and care personnel with low service quality should be promptly dismissed. The evaluation should focus on the satisfaction of the care recipients in the community. The evaluation content may include service process, service quantity, service quality and satisfaction with care plan, etc. Then, a comprehensive overall service satisfaction evaluation should be conducted. Finally, according to the evaluation results combined with the needs investigation of the care recipients in the community, the care services in the community are adjusted accordingly. Dynamic adjustment or annual adjustment can be decided according to the actual situation of each region and community.

## 4. Discussion

In 2016, China started the pilot work of the LTCI system. However, China’s LTC mainly relies on care service institutions at present, and the development of community nursing and institutional nursing is extremely unbalanced. Taking Nantong city as an example, by the end of 2021, the number of beds in care service institutions will exceed 96% of the city’s designated beds, while there are only 3 hospitals and community health service centers providing care services, and the number of beds will account for less than 5% [28].

The traditional Chinese family culture makes people more willing to receive LTC services at home. Rather than living in LTC service institutions, the development of community care can not only satisfy people’s desire to care for the elderly at home, but also effectively share the pressure of home care for family members. Therefore, this paper draws on the experience of establishing the ICCS in Japan, and puts forward relevant suggestions for the development of community care in China, expects that China’s LTCI can also increase the emphasis on community care during the pilot process, enables home care, institutional care, and community care to develop together and share the responsibility for LTC. The most fundamental of which is to strengthen institutional guarantees, China can establish LTCI-specific laws or regulations to provide legal support for LTC services just like medical insurance. The supporting policies related to LTCI can also improve and standardize the behavior of the care industry; therefore, it is necessary to formulate LTCI-related management specifications as soon as possible such as the qualification and incentives for caregivers, the standardization of elderly care facilities, etc.

However, it should be noted that the LTCI system in Japan is not without its limitations, and faces many challenges. Two of the biggest issues are the sustainability of LTC funding and the shortage of nursing staff. The population is rapidly aging, and political turmoil and a natural disaster also burdened the country, and this has contributed to the potentially unsustainable problem of LTC funding. China’s aging development is rapid, so the issue of LTC funding also needs to be paid great attention to, and it may be necessary to seek better financing methods to ensure the sustainability of funds. In addition, the low income of nursing staff has also led to a shortage of nursing staff, which is why we suggest encouraging diverse subjects to participate in community care services. In addition, we believe that how to improve the social recognition and treatment of nursing staff needs further research.

## Figures and Tables

**Table 1:** The long-term care insurance system in Japan.

Country	Japan
**Laws**	Long-Term Care Insurance Act (2000)
**Management department**	Municipalities and Prefectures
**The subject of the service being provided**	For-profit corporations, non-profit organizations
**Beneficiaries**	Category I: the elderly aged 65 years and above Category II: people aged 40–64 years with disabilities
**Finance**	Half comes from taxes (25% from the Central Government, 12.5% from the prefectures, and 12.5% from the municipalities) and half comes from premium contributions.
**Service/Payment Content**	Institutional and domiciliary services
**Care Levels**	Seven care levels: two requiring support (levels 1 and 2) and five requiring long-term care (levels 1–5)

**Table 2:** The reforms of Japan’s long-term care insurance *.

Years	Contents
**2003**	Revision of the Category 1 premium, revision of long-term care fees
**2005**	(1) Enactment of the law to revise a part of the Long-term Care Insurance Law: amendments to LTC fees, premium rates, and portions of the Long-term Care Insurance Act (2) A review of facility benefit
**2008**	Strengthen government supervision: rectify nursing corruption and management system; strengthen the restraint mechanism of nursing institutions
**2011**	(1) Expand the content of nursing services (2) Improve the treatment of caregivers
**2014**	(1) Establishing the Community-based Integrated Care System: enriching services and making services more focused and efficient (2) Making Contribution Equitable: expanding reduction of premiums of people with low incomes, and reviewing co-payments
**2017**	(1) Promotion of initiatives for strengthening insurers’ function, etc., toward independence support and prevention of serious conditions (2) Promotion of coordination between medical care and long-term care (3) Promotion of initiatives to realize a regional cohesive society (4) Increasing co-payment rate to 30% for those with particularly high income among persons bearing 20% co-payment (5) Introducing income-based payment system of long-term care levy (changing from capitation-based payment system)

* Compiled from relevant information on the official website of the Japanese Ministry of Health, Labour and Welfare.

## Data Availability

The study did not report any data.
